# Supplementary material for: An in-solution snapshot of SARS-COV-2 main protease maturation process and inhibition
Source: Nat Commun. 2023 Mar 20;14:1545. doi: 10.1038/s41467-023-37035-5 (PMC10027274; doi:10.1038/s41467-023-37035-5)
Supplement: Supplementary file 1 — SUPPLEMENTARY INFO [file 41467_2023_37035_MOESM1_ESM.pdf]

Supplementary Material Information

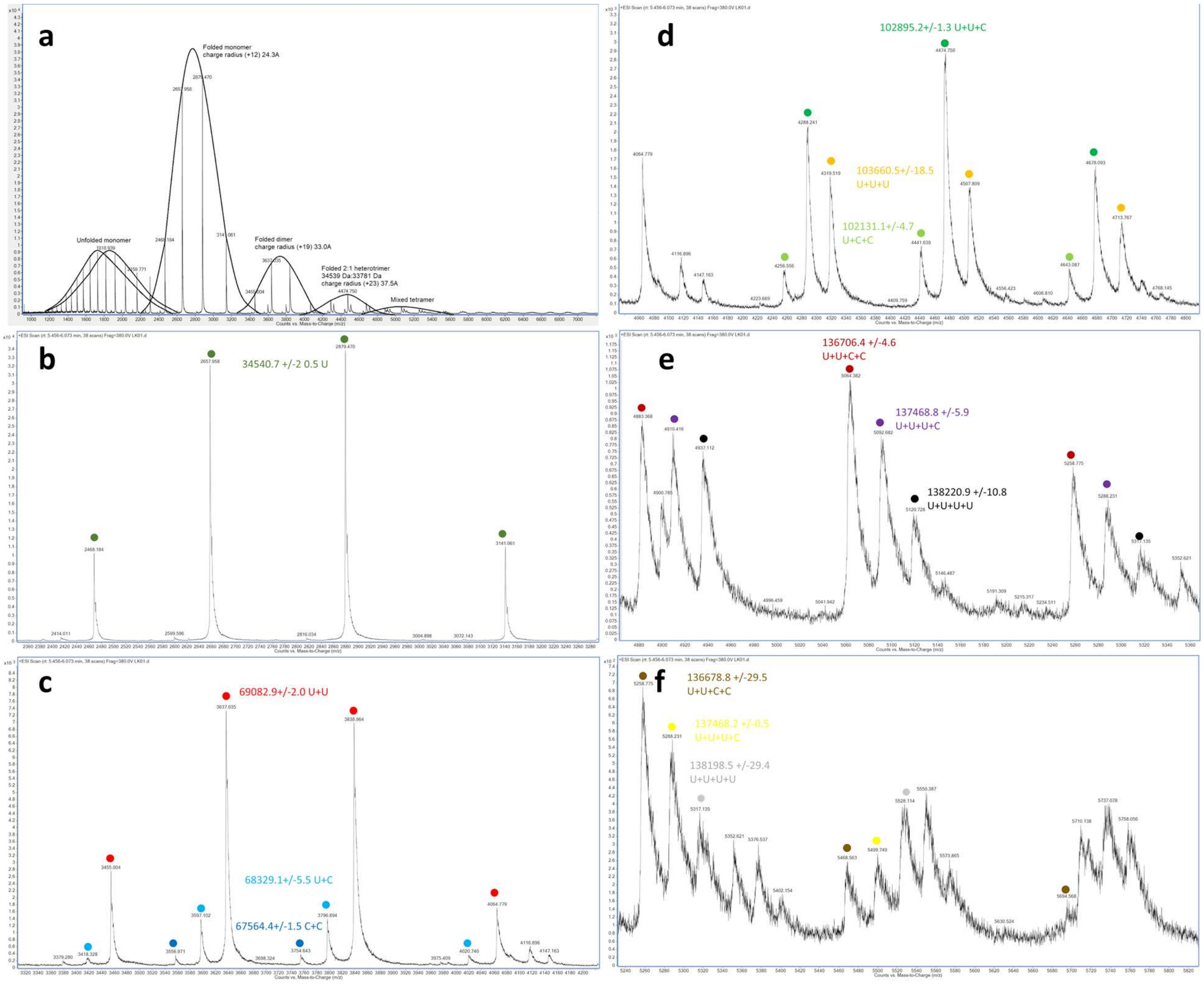

Supplementary Figure 1. Native mass spectroscopy of SARS-CoV-2 M^{pro} C145S data analysis. a. Complete profile from the sample 2, showing distinct oligomeric states. b. Analysis of primary peak containing folded monomers. c. Analysis of peaks containing folded dimers, showing that oligomers can be formed by all combinatory possibilities uncleaved (U) and cleaved (C) particles. d. Analysis of peaks containing folded trimers, showing that oligomers can be formed by all combinatory possibilities U and particles. e and f Analysis of peaks containing folded tetramers, showing that oligomers can be formed by all combinatory possibilities U and C particles.

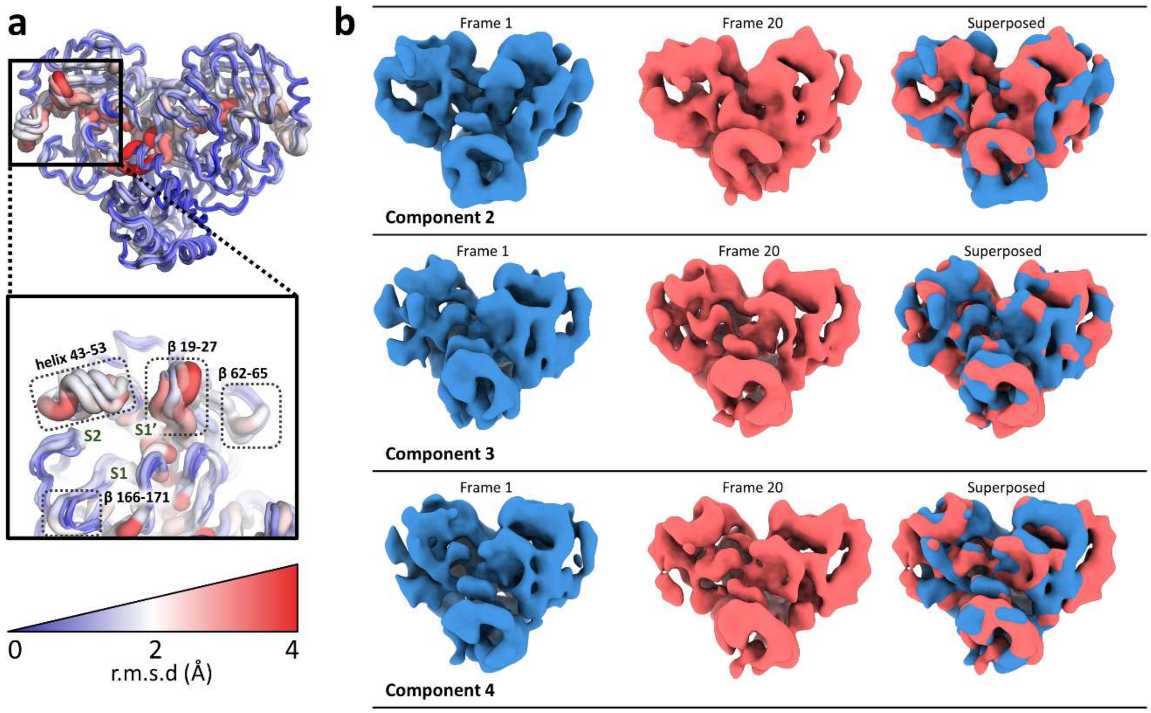

Supplementary Figure 2. 3DVA of SARS-CoV-2 M^{pro} C145S. a. Aligned models of M^{pro} that were morphed in distinct frames of 3DVA models, colored according to its r.m.s.d. The square box is highlighting the active site loops were we observed the largest r.m.s.d values. b. 3DVA maps of components 2, 3 and 4 showing first (blue), last (red) and superposed maps.

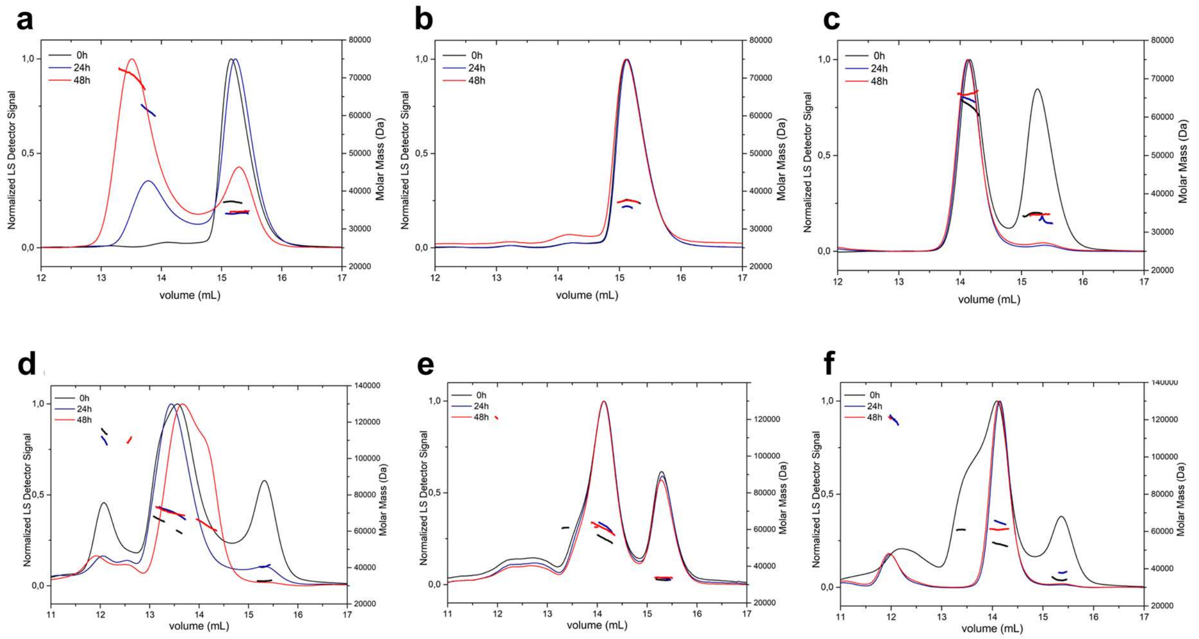

Supplementary Figure 3. SEC elution profiles of monomeric peak of C145S M^{pro}. a. SEC-MALS elution profiles of C145S M^{pro} monomers after 0h (black), 24h (blue) and 48h (red) incubation. b. SEC-MALS elution profiles of C145S M^{pro} monomers in presence of MAT-POS-e194df51-1 after 0h (black), 24h (blue) and 48h (red) incubation. c. SEC-MALS elution profiles of C145S M^{pro} monomers in presence of Nirmatrelvir after 0h (black), 24h (blue) and 48h (red) incubation. d. SEC-MALS elution profiles of C145S M^{pro} tetramers after 0h (black), 24h (blue) and 48h (red) incubation. e. SEC-MALS elution profiles of C145S M^{pro} tetramers in presence of MAT-POS-e194df51-1 after 0h (black), 24h (blue) and 48h (red) incubation. f. SEC-MALS elution profiles of C145S M^{pro} tetramers in presence of Nirmatrelvir after 0h (black), 24h (blue) and 48h (red) incubation. In SEC-MAL graphs, curves correspond to the change in the normalized scattered light intensity at 90° (lines) and calculated molar mass of the corresponding peak (dots) are given for each peak. Due the limits of resolution, trimers and tetramers were treated as tetramers. Graphs were plotted from individual SEC-MALS experiments (n=1), compared with multiple concentration controls.

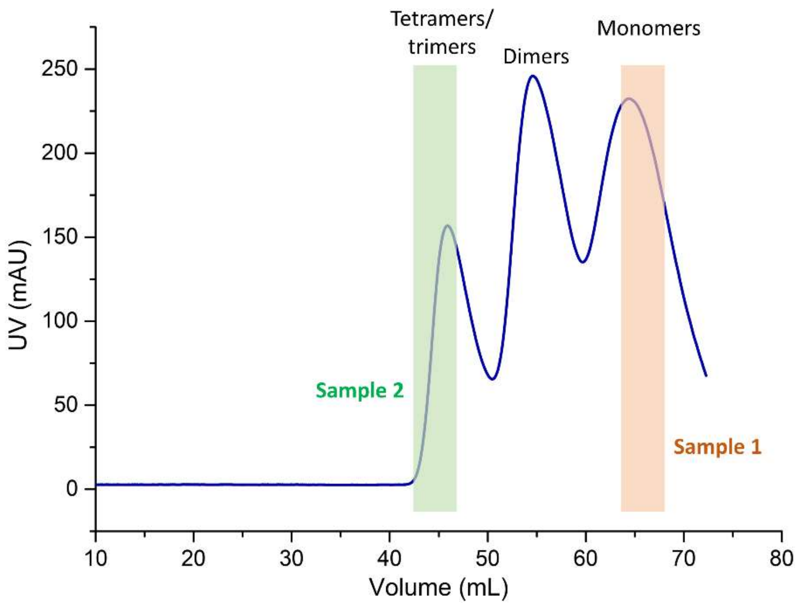

Supplementary Figure 4. Final size exclusion chromatography from SARS-CoV-2 M^{pro} C145S purification. Graph shows overlapping peaks of multiple oligomeric states. Colored areas demark fractions used for composing sample 1 (orange area) and sample 2 (green area). SDS-page shows sample 1 (left) and sample 2 (right), with molecular marker. Graphs were plotted of individual a size exclusion chromatography from SARS-CoV-2 experiment exemplary. Original SDS-page is available in Source data.

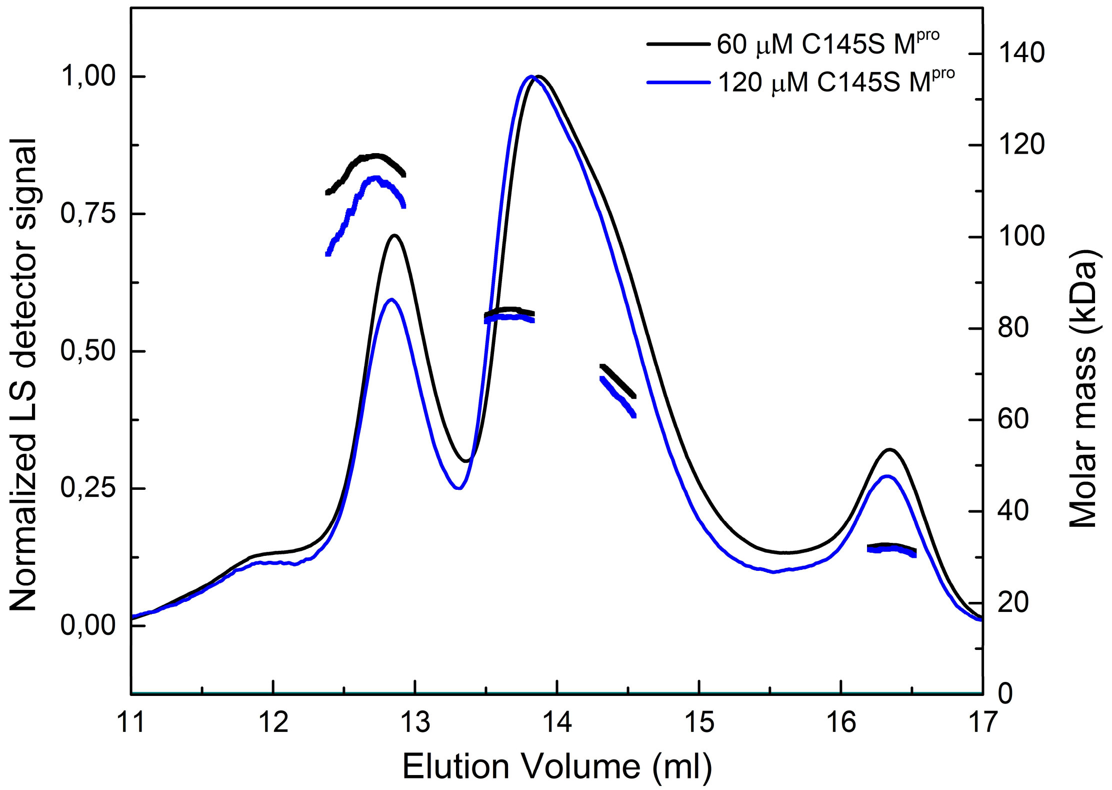

Supplementary Figure 5. SEC-MALS calibration showing two injections concentration of sample 2. In black, we see the injection of sample 2 at 60 μM, while in blue we see the injection of sample 2 at 120 μM. Graphs were plotted from individual SEC-MALS experiments in two concentrations.

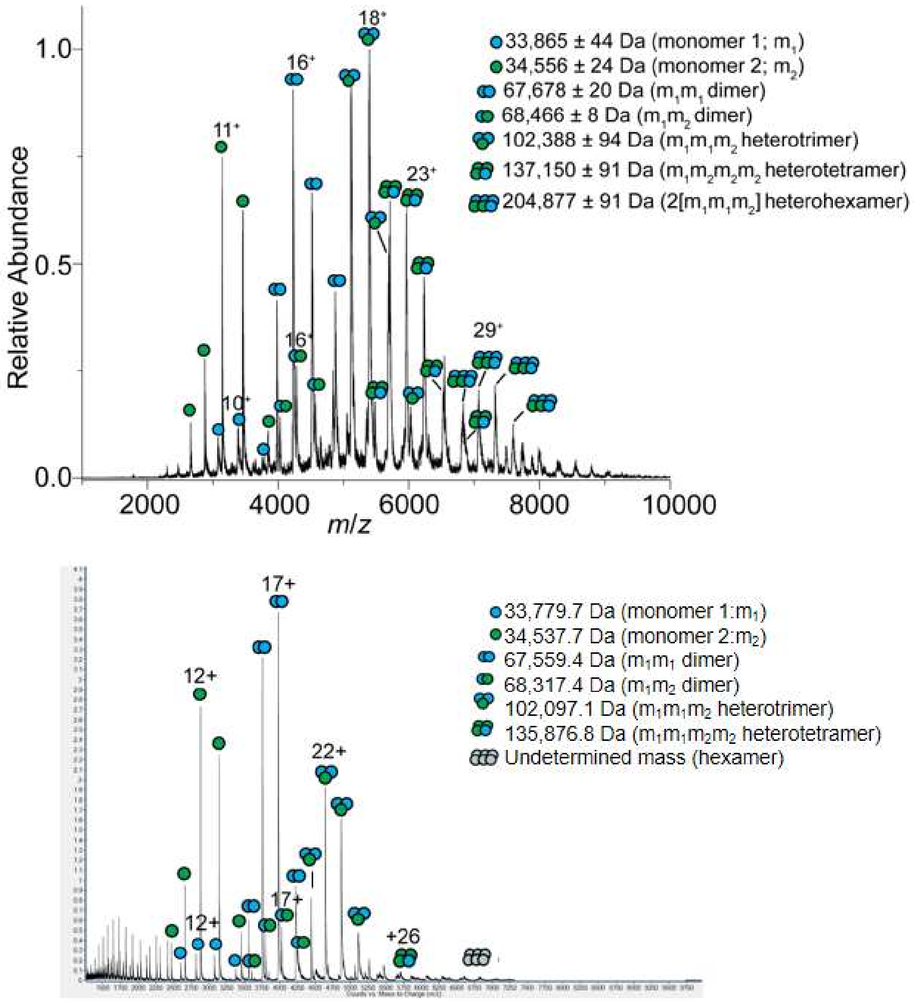

Supplementary Figure 6. Native mass spectrometry analysis of M^pro C145S tetrameric sample in two different instruments. At the top, native mass spectrometry using Orbitrap Q-Exactive UHMR instrument. At bottom, the data from main Figure 1, collected in the Agilent 1100 HPLC system, and plotted in the same style for comparison. Monomer peaks corresponding to the light and heavy forms of M^pro are labelled with blue and green circles, respectively. Molecular weights are green - 33,865 ± 44 Da; blue - 34,556 ± 24 Da. Homo- and hetero-oligomer peaks are labelled according to this color scheme, with the number of each colored circle corresponding to the oligomer assignment. Graphs were plotted from individual Native mass spectrometry experiments.

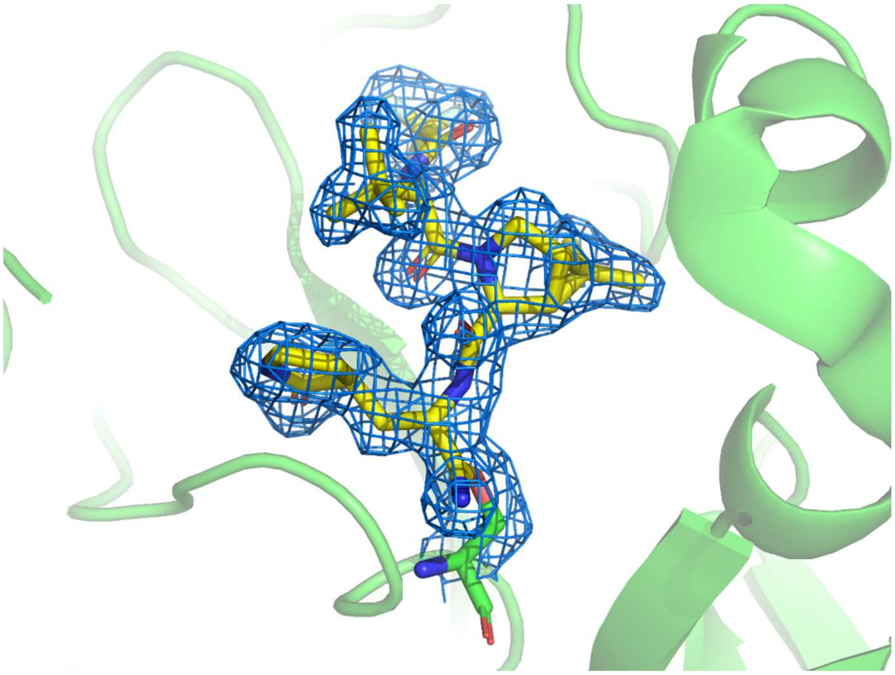

Supplementary Figure 7. C145S bound to Nirmatrelvir. Figure show electron-density from Nirmatrelvir bound to C145S M^{pro}. Electron density is represented as blue mesh and was calculated from 2Fo-Fc map with 1.0 σ. Nirmatrelvir (yellow) and S145 residue are show as sticks and M^{pro} as green cartoon. Maps and models are available in PDB under code 8EYJ.

Consortia Members and Affiliations

COVID Moonshot Consortium

NAME	INSTITUTION(S)	DEPARTMENT	ADDRESS	EMAIL
**MATTHEW C. ROBINSON**	PostEra Inc.	Technology	2 Embarcadero Center, 8th Floor, San Francisco, CA 94111, United States	matthew.robinson@postera.ai
**NIR LONDON**	The Weizmann Institute of Science	Chemical and Structural Biology	Hertzl 234 st. Rehovot, Israel, 7610001	nir.london@weizmann.ac.il
**EFRAT RESNICK**	The Weizmann Institute of Science	Chemical and Structural Biology	Hertzl 234 st. Rehovot, Israel, 7610001	efrat.resnick@weizmann.ac.il
**DANIEL ZAIDMANN**	The Weizmann Institute of Science	Chemical and Structural Biology	Hertzl 234 st. Rehovot, Israel, 7610001	daniel.zaidman@weizmann.ac.il
**PAUL GEHRTZ**	The Weizmann Institute of Science	Chemical and Structural Biology	Hertzl 234 st. Rehovot, Israel, 7610001	paul.gehrtz@weizmann.ac.il
**RAMBABU N. REDDI**	The Weizmann Institute of Science	Chemical and Structural Biology	Hertzl 234 st. Rehovot, Israel, 7610001	rambabu.reddi@weizmann.ac.il
**RONEN GABIZON**	The Weizmann Institute of Science	Chemical and Structural Biology	Hertzl 234 st. Rehovot, Israel, 7610001	ronen.gabizon@weizmann.ac.il
**HAIM BARR**	The Weizmann Institute of Science	Wohl Institute for Drug Discovery of the Nancy and Stephen Grand Israel National Center for Personalized Medicine	Hertzl 234 st. Rehovot, Israel, 7610001	haim.barr@weizmann.ac.il
**SHIRLY DUBERSTEIN**	The Weizmann Institute of Science	Wohl Institute for Drug Discovery of the Nancy and Stephen Grand Israel National Center for Personalized Medicine	Hertzl 234 st. Rehovot, Israel, 7610001	shirly.valter@weizmann.ac.il
**HADEER ZIDANE**	The Weizmann Institute of Science	Wohl Institute for Drug Discovery of the Nancy and Stephen Grand Israel National Center for Personalized Medicine	Hertzl 234 st. Rehovot, Israel, 7610001	hadeer.zidane@weizmann.ac.il

**KHRIESTO SHURRUSH**	The Weizmann Institute of Science	Wohl Institute for Drug Discovery of the Nancy and Stephen Grand Israel National Center for Personalized Medicine	Hertzl 234 st. Rehovot, Israel, 7610001	khriesto.shurrush@weizmann.ac.il
**GALIT COHEN**	The Weizmann Institute of Science	Wohl Institute for Drug Discovery of the Nancy and Stephen Grand Israel National Center for Personalized Medicine	Hertzl 234 st. Rehovot, Israel, 7610001	galit.cohen@weizmann.ac.il
**LEONARDO J. SOLMESKY**	The Weizmann Institute of Science	Wohl Institute for Drug Discovery of the Nancy and Stephen Grand Israel National Center for Personalized Medicine	Hertzl 234 st. Rehovot, Israel, 7610001	leonardo.solmesky@weizmann.ac.il
**ALPHA LEE**	PostEra Inc.; University of Cambridge			alpha.lee@postera.ai
**ANDREW JAJACK**	PostEra Inc.	Technology	2 Embarcadero Center, 8th Floor, San Francisco, CA 94111, United States	ajajack@gmail.com
**MILAN CVITKOVIC**	PostEra Inc.	Technology	2 Embarcadero Center, 8th Floor, San Francisco, CA 94111, United States	milan.cvitkovic@postera.ai
**JIN PAN**	PostEra Inc.	Technology	2 Embarcadero Center, 8th Floor, San Francisco, CA 94111, United States	jin.pan@postera.ai
**RUBY PAI**	PostEra Inc.	Technology	2 Embarcadero Center, 8th Floor, San Francisco, CA 94111, United States	ruby.pai@postera.ai
**EMILY GRACE RIPKA**	PostEra Inc.	Technology	2 Embarcadero Center, 8th Floor, San Francisco, CA 94111, United States	emily.ripka@postera.ai
**LUONG NGUYEN**	PostEra Inc.	Technology	2 Embarcadero Center, 8th Floor,	luong.nguyen@postera.ai

			San Francisco, CA 94111, United States	
**MIKHAIL SHAFEEV**	Enamine Ltd	Synthetic Chemistry	78 Chervonotkatska Street, 02094 Kyiv, Ukraine	m.shafeev@mail.enamine.net
**TATIANA MATVIIUK**	Enamine Ltd	Synthetic Chemistry	78 Chervonotkatska Street, 02094 Kyiv, Ukraine	t.matviyuk@enamine.net
**OLEG MICHURIN**	Enamine Ltd	Synthetic Chemistry	78 Chervonotkatska Street, 02094 Kyiv, Ukraine	o.michurin@enamine.net
**EUGENE CHERNYSHENKO**	Enamine Ltd	Synthetic Chemistry	78 Chervonotkatska Street, 02094 Kyiv, Ukraine	e.chernyshenko@mail.enamine.net
**VITALIY A. BILENKO**	Enamine Ltd	Synthetic Chemistry	78 Chervonotkatska Street, 02094 Kyiv, Ukraine	v.bilenko@mail.enamine.net
**SERHII O. KINAKH**	Enamine Ltd	Synthetic Chemistry	78 Chervonotkatska Street, 02094 Kyiv, Ukraine	s.kinakh@mail.enamine.net
**IVAN G. LOGVINENKO**	Enamine Ltd	Synthetic Chemistry	78 Chervonotkatska Street, 02094 Kyiv, Ukraine	i.logvinenko@mail.enamine.net
**KOSTIANTYN P. MELNYKOV**	Enamine Ltd	Synthetic Chemistry	78 Chervonotkatska Street, 02094 Kyiv, Ukraine	k.melnykov@mail.enamine.net
**VICTOR D. HULIAK**	Enamine Ltd	Synthetic Chemistry	78 Chervonotkatska Street, 02094 Kyiv, Ukraine	v.huliak@mail.enamine.net
**IGOR S. TSURUPA**	Enamine Ltd	Synthetic Chemistry	78 Chervonotkatska Street, 02094 Kyiv, Ukraine	i.tsurupa@mail.enamine.net
**MARIAN GORICHKO**	Taras Shevchenko National University of Kyiv	Organic Chemistry	Volodymyrska str. 64, 01033 Kyiv, Ukraine	417lab@gmail.com
**AARIF SHAIKH**	Sai Life Sciences	Synthetic Chemistry	Sai Life Sciences Limited, DS-7, ICICI Knowledge Park, Shameerpet, Hyderabad 500 078, Telangana, India, Phone No:	aarif.s@sailife.com

			+91 40 8418662201	
**JAKIR PINJARI**	Sai Life Sciences	Synthetic Chemistry	Sai Life Sciences Limited, DS-7, ICICI Knowledge Park, Shameerpet, Hyderabad 500 078, Telangana, India, Phone No: +91 40 8418662201	jakir.p@sailife.com
**VISHWANATH SWAMY**	Sai Life Sciences	Synthetic Chemistry	Sai Life Sciences Limited, DS-7, ICICI Knowledge Park, Shameerpet, Hyderabad 500 078, Telangana, India, Phone No: +91 40 8418662201	vishwanath.s@sailife.com
**MANEESH PINGLE**	Sai Life Sciences	Synthetic Chemistry	Sai Life Sciences Limited, DS-7, ICICI Knowledge Park, Shameerpet, Hyderabad 500 078, Telangana, India, Phone No: +91 40 8418662201	maneesh.p@sailife.com
**SARMA BVNBS**	Sai Life Sciences	Synthetic Chemistry	Sai Life Sciences Limited, DS-7, ICICI Knowledge Park, Shameerpet, Hyderabad 500 078, Telangana, India, Phone No: +91 40 8418662201	sarma.b@sailife.com
**ANTHONY AIMON**	Diamond Light Source Ltd; Research Complex at Harwell	Life Sciences	Diamond Light Source, Harwell Science and Innovation Campus, Didcot, OX11 0DE, United Kingdom; Research Complex at Harwell, Harwell Science and Innovation Campus, Didcot	anthony.aimon@diamond.ac.uk

			OX11 0FA, United Kingdom	
**FRANK VON DELFT**	Diamond Light Source Ltd; University of Oxford; Research Complex at Harwell; University of Johannesburg	Life Sciences	Diamond Light Source, Harwell Science and Innovation Campus, Didcot, OX11 0DE, United Kingdom; Centre for Medicine Discovery, Nuffield Department of Medicine, University of Oxford, Oxford, OX3 7DQ, United Kingdom; Research Complex at Harwell, Harwell Science and Innovation Campus, Didcot OX11 0FA, United Kingdom;Department of Biochemistry, University of Johannesburg, Auckland Park, 2006, South Africa	frank.von-delft@diamond.ac.uk
**DAREN FEARON**	Diamond Light Source Ltd; Research Complex at Harwell	Life Sciences	Diamond Light Source, Harwell Science and Innovation Campus, Didcot, OX11 0DE, United Kingdom; Research Complex at Harwell, Harwell Science and Innovation Campus, Didcot OX11 0FA, United Kingdom	daren.fearon@diamond.ac.uk
**LOUISE DUNNETT**	Diamond Light Source Ltd; Research Complex at Harwell	Life Sciences	Diamond Light Source, Harwell Science and Innovation Campus, Didcot, OX11 0DE, United	louise.dunnett@diamond.ac.uk

			Kingdom; Research Complex at Harwell, Harwell Science and Innovation Campus, Didcot OX11 0FA, United Kingdom	
**ALICE DOUANGAMATH**	Diamond Light Source Ltd; Research Complex at Harwell	Life Sciences	Diamond Light Source, Harwell Science and Innovation Campus, Didcot, OX11 0DE, United Kingdom; Research Complex at Harwell, Harwell Science and Innovation Campus, Didcot OX11 0FA, United Kingdom	alice.douangamath@diamond.ac.uk
**ALEX DIAS**	Diamond Light Source Ltd; Research Complex at Harwell	CEO	Diamond Light Source, Harwell Science and Innovation Campus, Didcot, OX11 0DE, United Kingdom; Research Complex at Harwell, Harwell Science and Innovation Campus, Didcot OX11 0FA, United Kingdom	alex.dias@diamond.ac.uk
**AILSA POWELL**	Diamond Light Source Ltd; Research Complex at Harwell	CEO	Diamond Light Source, Harwell Science and Innovation Campus, Didcot, OX11 0DE, United Kingdom; Research Complex at Harwell, Harwell Science and Innovation Campus, Didcot OX11 0FA, United Kingdom	ailsa.powell@diamond.ac.uk

**JOSE BRANDAO NETO**	Diamond Light Source Ltd; Research Complex at Harwell	Life Sciences	Diamond Light Source, Harwell Science and Innovation Campus, Didcot, OX11 0DE, United Kingdom; Research Complex at Harwell, Harwell Science and Innovation Campus, Didcot OX11 0FA, United Kingdom	jose.brandao-neto@diamond.ac.uk
**RACHAEL SKYNER**	Diamond Light Source Ltd; Research Complex at Harwell	Life Sciences	Diamond Light Source, Harwell Science and Innovation Campus, Didcot, OX11 0DE, United Kingdom; Research Complex at Harwell, Harwell Science and Innovation Campus, Didcot OX11 0FA, United Kingdom	rachael.skyner@diamond.ac.uk
**WARREN THOMPSON**	Diamond Light Source Ltd; Research Complex at Harwell	Life Sciences	Diamond Light Source, Harwell Science and Innovation Campus, Didcot, OX11 0DE, United Kingdom; Research Complex at Harwell, Harwell Science and Innovation Campus, Didcot OX11 0FA, United Kingdom	warren.thompson@diamond.ac.uk
**TYLER GORRIE-STONE**	Diamond Light Source Ltd; Research Complex at Harwell	Life Sciences	Diamond Light Source, Harwell Science and Innovation Campus, Didcot, OX11 0DE, United Kingdom; Research Complex at Harwell, Harwell Science and	tyler.gorrie-stone@diamond.ac.uk

			Innovation Campus, Didcot OX11 0FA, United Kingdom	
**MARTIN WALSH**	Diamond Light Source Ltd; Research Complex at Harwell	Life Sciences	Diamond Light Source, Harwell Science and Innovation Campus, Didcot, OX11 0DE, United Kingdom; Research Complex at Harwell, Harwell Science and Innovation Campus, Didcot OX11 0FA, United Kingdom	martin.walsh@diamond.ac.uk
**DAVID OWEN**	Diamond Light Source Ltd; Research Complex at Harwell	Life Sciences	Diamond Light Source, Harwell Science and Innovation Campus, Didcot, OX11 0DE, United Kingdom; Research Complex at Harwell, Harwell Science and Innovation Campus, Didcot OX11 0FA, United Kingdom	david.owen@diamond.ac.uk
**PETRA LUKACIK**	Diamond Light Source Ltd; Research Complex at Harwell	Life Sciences	Diamond Light Source, Harwell Science and Innovation Campus, Didcot, OX11 0DE, United Kingdom; Research Complex at Harwell, Harwell Science and Innovation Campus, Didcot OX11 0FA, United Kingdom	petra.lukacik@diamond.ac.uk
**CLAIRE STRAIN-DAMERELL**	Diamond Light Source Ltd; Research Complex at Harwell	Life Sciences	Diamond Light Source, Harwell Science and Innovation Campus, Didcot, OX11 0DE, United Kingdom;	claire.strain-damerell@diamond.ac.uk

			Research Complex at Harwell, Harwell Science and Innovation Campus, Didcot OX11 0FA, United Kingdom	
**HALINA MIKOLAJEK**	Diamond Light Source Ltd; Research Complex at Harwell	Life Sciences	Diamond Light Source, Harwell Science and Innovation Campus, Didcot, OX11 0DE, United Kingdom; Research Complex at Harwell, Harwell Science and Innovation Campus, Didcot OX11 0FA, United Kingdom	halina.mikolajek@diamond.ac.uk
**SAM HORRELL**	Diamond Light Source Ltd; Research Complex at Harwell	Life Sciences	Diamond Light Source, Harwell Science and Innovation Campus, Didcot, OX11 0DE, United Kingdom; Research Complex at Harwell, Harwell Science and Innovation Campus, Didcot OX11 0FA, United Kingdom	sam.horrell@diamond.ac.uk
**LIZBÉ KOEKEMOER**	University of Oxford	Nuffield Department of Medicine	Centre for Medicines Discovery, University of Oxford, ORCRB, Roosevelt Drive, Oxford, Oxon, OX3 7DQ, UK	lizbe.koekemoer@cmd.ox.ac.uk
**TOBIAS KROJER**	University of Oxford	Nuffield Department of Medicine	Centre for Medicines Discovery, University of Oxford, ORCRB, Roosevelt Drive, Oxford, Oxon, OX3 7DQ, UK	tobias.krojer@cmd.ox.ac.uk

**MIKE FAIRHEAD**	University of Oxford	Nuffield Department of Medicine	Centre for Medicines Discovery, University of Oxford, ORCRB, Roosevelt Drive, Oxford, Oxon, OX3 7DQ, UK	michael.fairhead@cmd.ox.ac.uk
**ELIZABETH M. MACLEAN**	University of Oxford	Nuffield Department of Medicine	Centre for Medicines Discovery, University of Oxford, ORCRB, Roosevelt Drive, Oxford, Oxon, OX3 7DQ, UK	beth.maclean@cmd.ox.ac.uk
**ANDREW THOMPSON**	University of Oxford	Nuffield Department of Medicine	Centre for Medicines Discovery, University of Oxford, ORCRB, Roosevelt Drive, Oxford, Oxon, OX3 7DQ, UK	andrew.thompson@cmd.ox.ac.uk
**CONOR FRANCIS WILD**	University of Oxford	Nuffield Department of Medicine	Centre for Medicines Discovery, University of Oxford, ORCRB, Roosevelt Drive, Oxford, Oxon, OX3 7DQ, UK	conor.wild@cmd.ox.ac.uk
**MIHAELA D. SMILOVA**	University of Oxford	Nuffield Department of Medicine	Centre for Medicines Discovery, University of Oxford, ORCRB, Roosevelt Drive, Oxford, Oxon, OX3 7DQ, UK	mihaela.smilova@cmd.ox.ac.uk
**NATHAN WRIGHT**	University of Oxford	Nuffield Department of Medicine	Centre for Medicines Discovery, University of Oxford, ORCRB, Roosevelt Drive, Oxford, Oxon, OX3 7DQ, UK	nathan.wright@cmd.ox.ac.uk
**ANNETTE VON DELFT**	University of Oxford	Nuffield Department of Medicine	Centre for Medicines Discovery, University of Oxford, ORCRB, Roosevelt Drive,	annette.vondelft@cmd.ox.ac.uk

			Oxford, Oxon, OX3 7DQ, UK	
**CARINA GILEADI**	University of Oxford	Nuffield Department of Medicine	Centre for Medicines Discovery, University of Oxford, ORCRB, Roosevelt Drive, Oxford, Oxon, OX3 7DQ, UK	carina.gileadi@cmd.ox.ac.uk
**VICTOR L. RANGEL**	School of Pharmaceutical Sciences of Ribeirao Preto	Pharmaceutical Sciences	Avenida do Café, s/nºRibeirão Preto - SP/CEP 14040-903	victor.rangel@usp.br
**CHRIS SCHOFIELD**	University of Oxford	Department of Chemistry	Chemistry Research Laboratory, 12 Mansfield Rd, Oxford OX1 3TA, United Kingdom	christopher.schofield@chem.ox.ac.uk
**EIDARUS SALAH**	University of Oxford	Department of Chemistry	Chemistry Research Laboratory, 12 Mansfield Rd, Oxford OX1 3TA, United Kingdom	eidarus.salah@chem.ox.ac.uk
**TIKA R. MALLA**	University of Oxford	Department of Chemistry	Chemistry Research Laboratory, 12 Mansfield Rd, Oxford OX1 3TA, United Kingdom	tika.malla@chem.ox.ac.uk
**ANTHONY TUMBER**	University of Oxford	Department of Chemistry	Chemistry Research Laboratory, 12 Mansfield Rd, Oxford OX1 3TA, United Kingdom	anthony.tumber@chem.ox.ac.uk
**TOBIAS JOHN**	University of Oxford	Department of Chemistry	Chemistry Research Laboratory, 12 Mansfield Rd, Oxford OX1 3TA, United Kingdom	tobias.john@chem.ox.ac.uk
**IOANNIS VAKONAKIS**	University of Oxford	Department of Biochemistry	South Parks Road, Oxford OX1 3QU	ioannis.vakonakis@bioch.ox.ac.uk
**ANASTASSIA L. KANTSADI**	University of Oxford	Department of Biochemistry	South Parks Road, Oxford OX1 3QU	anastassia.kantsadi@bioch.ox.ac.uk
**NICOLE ZITZMANN**	University of Oxford	Department of Biochemistry	Oxford Glycobiology Institute; South Parks Road, Oxford OX1 3QU	nicole.zitzmann@bioch.ox.ac.uk

**JULIANE BRUN**	University of Oxford	Department of Biochemistry	Oxford Glycobiology Institute; South Parks Road, Oxford OX1 3QU	juliane.brun@path.ox.ac.uk
**J. L. KIAPPES**	University of Oxford	Department of Biochemistry	Oxford Glycobiology Institute; South Parks Road, Oxford OX1 3QU	john.kiappes@bioch.ox.ac.uk
**MICHELLE HILL**	University of Oxford	Department of Biochemistry	Oxford Glycobiology Institute; South Parks Road, Oxford OX1 3QU	michelle.hill@bioch.ox.ac.uk
**KAROLINA D WITT**	University of Oxford	Department of Biochemistry	Oxford Glycobiology Institute; South Parks Road, Oxford OX1 3QU	karolina.witt@bioch.ox.ac.uk
**DOMINIC S ALONZI**	University of Oxford	Department of Biochemistry	Oxford Glycobiology Institute; South Parks Road, Oxford OX1 3QU	dominic.alonzi@bioch.ox.ac.uk
**LAETITIA L MAKOWER**	University of Oxford	Department of Biochemistry	Oxford Glycobiology Institute; South Parks Road, Oxford OX1 3QU	lettymakower@gmail.com
**FINNY S. VARGHESE**	Radboud University Medical Center	Department of Medical Microbiology	Radboud Institute for Molecular Life Sciences, Radboud University Medical Center, Geert Grooteplein Zuid 28, 6525 GA Nijmegen, The Netherlands	F.Varghese@uniqure.com
**GIJS J. OVERHEUL**	Radboud University Medical Center	Department of Medical Microbiology	Radboud Institute for Molecular Life Sciences, Radboud University Medical Center, Geert Grooteplein Zuid 28, 6525 GA Nijmegen, The Netherlands	Gijs.Overheul@radboudumc.nl
**PASCAL MIESEN**	Radboud University Medical Center	Department of Medical Microbiology	Radboud Institute for Molecular Life Sciences, Radboud University Medical	pascal.miesen@radboudumc.nl

			Center, Geert Grooteplein Zuid 28, 6525 GA Nijmegen, The Netherlands	
**RONALD P. VAN RIJ**	Radboud University Medical Center	Department of Medical Microbiology	Radboud Institute for Molecular Life Sciences, Radboud University Medical Center, Geert Grooteplein Zuid 28, 6525 GA Nijmegen, The Netherlands	ronald.vanrij@radboudumc.nl
**JITSKE JANSEN**	Radboud University Medical Center	Department of Pathology	Radboud Institute for Molecular Life Sciences, Radboud University Medical Center, Geert Grooteplein Zuid 28, 6525 GA Nijmegen, The Netherlands	Jitske.Jansen@radboudumc.nl
**BART SMEETS**	Radboud University Medical Center	Department of Pathology	Radboud Institute for Molecular Life Sciences, Radboud University Medical Center, Geert Grooteplein Zuid 28, 6525 GA Nijmegen, The Netherlands	Bart.Smeets@radboudumc.nl
**SUSANA TOMÁSIO**	Collaborative Drug Discovery		20 Station Rd, Cambridge, CB1 2JD, UK	Stomasio@collaborativedrug.com
**CHARLIE WEATHERALL**	Collaborative Drug Discovery		1633 Bayshore Hwy, Burlingame, CA 94010, USA	cweatherall@collaborativedrug.com
**MARIANA VASCHETTO**	Collaborative Drug Discovery		20 Station Rd, Cambridge CB1 2JD, UK	mariana@collaborativedrug.com
**HANNAH BRUCE MACDONALD**	Memorial Sloan Kettering Cancer Center	Computational and Systems Biology Program	1275 York Ave, New York, NY 10065	hannah.brucemacdonald@choderalab.org
**JOHN D. CHODERA**	Memorial Sloan Kettering Cancer Center	Computational and Systems Biology Program	Memorial Sloan Kettering Cancer Center	john.chodera@choderalab.org
**DOMINIC RUFA**	Memorial Sloan Kettering Cancer Center	Memorial Sloan Kettering Cancer Center	1275 York Ave, New York, NY 10065	dominic.rufa@choderalab.org

**MATTHEW WITTMANN**	Memorial Sloan Kettering Cancer Center	Computational and Systems Biology Program	1275 York Ave, New York, NY 10065	matt.wittmann@choderalab.org
**MELISSA L. BOBY**	Memorial Sloan Kettering Cancer Center;Weil Cornell Medical College	Weil Cornell Graduate School of Medical Sciences	1275 York Ave, New York, NY 10065	melissa.boby@choderalab.org
**MICHAEL HENRY**	Memorial Sloan Kettering Cancer Center	Memorial Sloan Kettering Cancer Center	Memorial Sloan Kettering Cancer Center	mike.henry@choderalab.org
**WILLIAM G. GLASS**	Memorial Sloan Kettering Cancer Center	Computational and Systems Biology Program	1275 York Ave, New York, NY 10065	william.glass@choderalab.org
**PETER K. EASTMAN**	Department of Bioengineering until Sept. 1, then Department of Chemistry	Stanford University	11 Mill Site Road, Scotts Valley, CA 95066	peastman@stanford.edu
**JOSEPH E. COFFLAND**	N/A	Cauldron Development LLC	523 6th Street, Petaluma, CA 94952	joseph@caulrondevelopment.com
**DAVID L. DOTSON**	N/A	Datryllic LLC	280 W. McKinley St., #314, Phoenix AZ, 85003	david@datryllic.com
**ED J. GRIFFEN**	MedChemica Ltd	Research and Development	Alderley Park, Macclesfield, Cheshire. SK10 4TG UK.	ed.griffen@medchemica.com
**WILLAM MCCORKINDALE**	University of Cambridge	Cavendish Laboratory	19 JJ Thomson Avenue, Cambridge, CB3 0HE UK	
**AARON MORRIS**	PostEra Inc	CEO	2 Embarcadero Center, 8th Floor, San Francisco, CA 94111, United States	aaron.morris@postera.ai
**ROBERT GLEN**	University of Cambridge	Department of Chemistry	Lensfield Road, Cambridge, CB21EW, United Kingdom	rcg28@cam.ac.uk
**JASON COLE**	Cambridge Crystallographic Datacentre		12 Union Road, Cambridge, CB2 1EZ, United Kingdom	cole@ccdc.cam.ac.uk
**RICHARD FOSTER**	University of Leeds	School of Chemistry	Leeds, LS2 9JT, United Kingdom	R.Foster@leeds.ac.uk
**HOLLY FOSTER**	University of Leeds	School of Chemistry	Leeds, LS2 9JT, United Kingdom	cm13hf@leeds.ac.uk
**MARK CALMIANO**	UCB		216 Bath Road, Slough, SL1 3WE, United Kingdom	mark.calmiano@ucb.com
**RACHAEL E. TENNANT**	Lhasa Limited		Granary Wharf House, 2 Canal	Rachael.Tennant@lhasalimited.org

			Wharf, Holbeck, Leeds LS11 5PS, United Kingdom	
**JAG HEER**	UCB		216 Bath Road, Slough, SL1 3WE, United Kingdom	jag.heer@ucb.com
**JIYE SHI**	UCB		216 Bath Road, Slough, SL1 3WE, United Kingdom	jiye.shi@ucb.com
**ERIC JNOFF**	UCB		Chemin du Foriest, 1420 Braine-l'Alleud, Belgium	eric.jnoff@ucb.com
**MATTHEW F.D. HURLEY**	Temple University	Department of Chemistry	1901 N 13th st. Philadelphia, PA 19122	matthew.hurley@temple.edu
**BRUCE A. LEFKER**	Thames Pharma Partners LLC		1 Conrad Street, Mystic, CT 06355, USA	bruce.lefker@thamespharmapartners.com
**RALPH P. ROBINSON**	Thames Pharma Partners LLC		1 Conrad Street, Mystic, CT 06355, USA	ralph.p.robinson@thamespharmapartners.com
**CHARLINE GIROUD**	University of Oxford	Nuffield Department of Medicine	Centre for Medicines Discovery & Target Discovery Institute, University of Oxford, Oxford OX3 7FZ, UK	charline.giroud@cmd.ox.ac.uk
**JAMES BENNETT**	University of Oxford	Nuffield Department of Medicine	Centre for Medicines Discovery & Target Discovery Institute, University of Oxford, Oxford OX3 7FZ, UK	james.bennett@cmd.ox.ac.uk
**OLEG FEDOROV**	University of Oxford	Nuffield Department of Medicine	Centre for Medicines Discovery & Target Discovery Institute, University of Oxford, Oxford OX3 7FZ, UK	oleg.fedorov@cmd.ox.ac.uk

**ST PATRICK REID**	Department of Pathology and Microbiology	University of Nebraska Medical Centre	985900 Nebraska Medical Center, Omaha, NE 68198-5900, USA	patrick.reid@unmc.edu
**MELODY JANE MORWITZER**	Department of Pathology and Microbiology	University of Nebraska Medical Centre	985900 Nebraska Medical Center, Omaha, NE 68198-5900, USA	
**LISA COX**	Life Compass Consulting Ltd	Project Management	26 Millers Meadow, Rainow, Macclesfield. SK10 5UE	lisa@lifecompassconsulting.co.uk
**GARRETT M. MORRIS**	University of Oxford	Department of Statistics	24-29 St Giles', Oxford OX1 3LB, UK	morris@stats.ox.ac.uk
**MATTEO FERLA**	University of Oxford	Nuffield Department of Medicine	Wellcome Centre for Human Genetics, Roosevelt Drive, Oxford OX3 7BN, UK	matteo@well.ox.ac.uk
**DEMETRI MOUSTAKAS**	Relay Therapeutics			dmoustakas@relaytx.com
**TIM DUDGEON**	Informatics Matters			tdudgeon@informaticsmatters.com
**VLADIMÍR PŠENÁK**	M2M solutions, s.r.o		Vysokoškolákov 1757/1, 010 01 Žilina, Slovenská republika	vladimir.psenak@m2ms.sk
**BORIS KOVAR**	M2M solutions, s.r.o		Vysokoškolákov 1757/1, 010 01 Žilina, Slovenská republika	boris.kovar@m2ms.sk
**VINCENT VOELZ**	Temple University			voelz@temple.edu
**ANNA CARBERY**	University of Oxford;Diamond Light Source	Department of Statistics	24-29 St Giles', Oxford OX1 3LB, UK; Diamond Light Source, Harwell Science and Innovation Campus, Didcot, OX11 0DE, United Kingdom	anna.carbery@lmh.ox.ac.uk
**ALESSANDRO CONTINI**	University of Milan	DISFARM - Sezione di Chimica Generale e Organica "Alessandro Marchesini"	Via Venezian, 21 20133 Milano	Alessandro.Contini@unimi.it
**AUSTIN CLYDE**	Argonne National Laboratory			aclyde@anl.gov

**AMIR BEN-SHMUEL**	Israel Institution of Biological Research		Ness-Ziona, Israel	amirb@iibr.gov.il
**ASSA SITTNER**	Israel Institution of Biological Research		Ness-Ziona, Israel	assas@iibr.gov.il
**BOAZ POLITI**	Israel Institution of Biological Research		Ness-Ziona, Israel	boazp@iibr.gov.il
**EINAT B. VITNER**	Israel Institution of Biological Research		Ness-Ziona, Israel	einatv@iibr.gov.il
**ELAD BAR-DAVID**	Israel Institution of Biological Research		Ness-Ziona, Israel	eladb@iibr.gov.il
**HADAS TAMIR**	Israel Institution of Biological Research		Ness-Ziona, Israel	hadast@iibr.gov.il
**HAGIT ACHDOUT**	Israel Institution of Biological Research		Ness-Ziona, Israel	hagita@iibr.gov.il
**HAIM LEVY**	Israel Institution of Biological Research		Ness-Ziona, Israel	haiml@iibr.gov.il
**ITAI GLINERT**	Israel Institution of Biological Research		Ness-Ziona, Israel	itaig@iibr.gov.il
**NIR PARAN**	Israel Institution of Biological Research		Ness-Ziona, Israel	nirp@iibr.gov.il
**NOAM EREZ**	Israel Institution of Biological Research		Ness-Ziona, Israel	noame@iibr.gov.il
**REUT PUNI**	Israel Institution of Biological Research		Ness-Ziona, Israel	reutp@iibr.gov.il
**SHARON MELAMED**	Israel Institution of Biological Research		Ness-Ziona, Israel	sharonm@iibr.gov.il
**SHAY WEISS**	Israel Institution of Biological Research		Ness-Ziona, Israel	shayw@iibr.gov.il
**TOMER ISRAELY**	Israel Institution of Biological Research		Ness-Ziona, Israel	tomeri@iibr.gov.il
**YFAT YAHALOM-RONEN**	Israel Institution of Biological Research		Ness-Ziona, Israel	yfatyr@iibr.gov.il
**ADAM SMALLEY**	UCB		216 Bath Road, Slough, SL1 3WE, United Kingdom	adam.smalley@ucb.com
**VLADAS OLEINIKOVAS**	UCB		216 Bath Road, Slough, SL1 3WE, United Kingdom	vladas.oleinikovas@ucb.com

**JOHN SPENCER**	University of Sussex	Department of Chemistry, School of Life Sciences,	University of Sussex Falmer, Brighton, East Sussex, BN1 9QJ, UK.	J.Spencer@sussex.ac.uk
**PETER W. KENNY**			Berwick-on-Sea, North Coast Road, Blanchisseuse, Saint George, Trinidad and Tobago	pwk.pub.2008@gmail.com
**WALTER WARD**	Walter Ward Consultancy & Training	Enzymology		wal.ward@hotmail.co.uk
**EMMA CATTERMOLE**	University of Oxford	Department of Biochemistry	South Parks Road, Oxford OX1 3QU	emma.cattermole@bioch.ox.ac.uk
**LORI FERRINS**	Northeastern University	Department of Chemistry and Chemical Biology		l.ferrins@northeastern.edu
**CHARLES J. EYERMANN**	Northeastern University	Department of Chemistry and Chemical Biology		c.eyermann@northeastern.edu
**BRUCE F. MILNE**	University of Coimbra and University of Aberdeen	CFisUC, Department of Physics, University of Coimbra; Department of Chemistry, University of Aberdeen, Old Aberdeen, AB24 3UE, Scotland, UK	Rua Larga, 3004-516 Coimbra, Portugal ; Old Aberdeen, AB24 3UE Scotland, UK	bfmilne@uc.pt ; bruce.milne@abdn.ac.uk
**ANDRE S. GODOY**	University of Sao Paulo	Sao Carlos Institute of Physics	Av. Joao Dagnone, 1100 , Sao Carlos, 13563-120, Brazil	andregodoy@ifsc.usp.br
**GABRIELA D. NOSKE**	University of Sao Paulo	Sao Carlos Institute of Physics	Av. Joao Dagnone, 1100 , Sao Carlos, 13563-120, Brazil	gabriela.noske@usp.br
**GLAUCIUS OLIVA**	University of Sao Paulo	Sao Carlos Institute of Physics	Av. Joao Dagnone, 1100 , Sao Carlos, 13563-120, Brazil	oliva@ifsc.usp.br
**RAFAELA S. FERNANDES**	University of Sao Paulo	Sao Carlos Institute of Physics	Av. Joao Dagnone, 1100 , Sao Carlos, 13563-120, Brazil	rafaela.fernandes@usp.br
**ALINE M. NAKAMURA**	University of Sao Paulo	Sao Carlos Institute of Physics	Av. Joao Dagnone, 1100 , Sao Carlos, 13563-120, Brazil	aline.nakamura@ifsc.usp.br
**VICTOR O. GAWRILJUK**	University of Sao Paulo	Sao Carlos Institute of Physics	Av. Joao Dagnone, 1100 ,	victor.gawriljuk.oliveira@usp.br

			Sao Carlos, 13563-120, Brazil	
**KRIS M. WHITE**	Icahn School of Medicine at Mount Sinai	Icahn School of Medicine	1 Gustave L. Levy Pl., New York, NY 10021	kris.white@mssm.edu
**BRIANA L. MCGOVERN**	Icahn School of Medicine at Mount Sinai	Icahn School of Medicine	1 Gustave L. Levy Pl., New York, NY 10021	briana.mcgovern@mssm.edu
**ROMEL ROSALES**	Icahn School of Medicine at Mount Sinai	Icahn School of Medicine	1 Gustave L. Levy Pl., New York, NY 10021	romel.rosales@mssm.edu
**ADOLFO GARCIA-SASTRE**	Icahn School of Medicine at Mount Sinai	1. Department of Microbiology, 2. Global Health and Emerging Pathogens Institute, 3. Department of Medicine, Division of Infectious Diseases, 4. The Tisch Cancer Institute, 5. Department of Pathology, Molecular and Cell-Based Medicine	1468 Madison Avenue, New York, NY 10029, USA	adolfo.garcia-sastre@mssm.edu
**DANIEL CARNEY**	Takeda Development Center Americas, Inc.		6381 Towne Centre Drive, San Diego, CA 92121, USA	daniel.carney@takeda.com
**EDCON CHANG**	Takeda Development Center Americas, Inc.		6381 Towne Centre Drive, San Diego, CA 92121, USA	edcon.chang@takeda.com
**KUMAR SINGH SAIKATENDU**	Takeda Development Center Americas, Inc.		6381 Towne Centre Drive, San Diego, CA 92121, USA	kumar.saikatendu@takeda.com
**LAURA VANGEEL**	Katholieke Universiteit Leuven	Department of Microbiology, Immunology and Transplantation	Rega Institute for Medical Research, Herestraat 49, Laboratory of Virology and Chemotherapy, Leuven, Belgium	laura.vangeel@kuleuven.be
**JOHAN NEYTS**	Katholieke Universiteit Leuven	Department of Microbiology, Immunology and Transplantation	Rega Institute for Medical Research, Herestraat 49,	johan.neyts@kuleuven.be

			Laboratory of Virology and Chemotherapy, Leuven, Belgium	
**KIM DONCKERS**	Katholieke Universiteit Leuven	Department of Microbiology, Immunology and Transplantation	Rega Institute for Medical Research, Herestraat 49, Laboratory of Virology and Chemotherapy, Leuven, Belgium	kim.donckers@kuleuven.be
**DIRK JOCHMANS**	Katholieke Universiteit Leuven	Department of Microbiology, Immunology and Transplantation	Rega Institute for Medical Research, Herestraat 49, Laboratory of Virology and Chemotherapy, Leuven, Belgium	dirk.jochmans@kuleuven.be
**STEVEN DE JONGHE**	Katholieke Universiteit Leuven	Department of Microbiology, Immunology and Transplantation	Rega Institute for Medical Research, Herestraat 49, Laboratory of Virology and Chemotherapy, Leuven, Belgium	steven.dejonghe@kuleuven.be
**GREGORY R. BOWMAN**	Washington University School of Medicine	Dept of Biochemistry & Molecular Biophysics	4523 Clayton Ave, St. Louis, MO 63110	g.bowman@wustl.edu
**BRUCE BORDEN**	Folding@home Consortium			borden.b@gmail.com
**SUKRIT SINGH**	Memorial Sloan Kettering Cancer Center	Computational and Systems Biology Program	1275 York Ave, New York, NY 10065	sukrit.singh@choderalab.org
**ANDREA VOLKAMER**	Charité Universitätsmediz in Berlin	In silico Toxicology and Structural Bioinformatics	Virchowweg 6, 10117 Berlin, Germany	andrea.volkamer@charite.de
**JAIME RODRIGUEZ-GUERRA**	Charité Universitätsmediz in Berlin	In silico Toxicology and Structural Bioinformatics	Virchowweg 6, 10117 Berlin, Germany	jaime.rogue@gmail.com
**GWEN FATE**	Thames Pharma Partners	Medicinal Chemistry	1 Conrad Street, Mystic, CT 06355, USA	g.fate@thamespharmapartners.com
**STORM HASSELL HART**	University of Sussex	Department of Chemistry, School of Life Sciences,	University of Sussex Falmer, Brighton, East Sussex, BN1 9QJ, UK.	S.Hassell-Hart@sussex.ac.uk

**VITALIY A. BILENKO**	Enamine Ltd	Medicinal Chemistry	78 Chervonotkatska Street, 02094 Kyiv, Ukraine	v.bilenko@mail.enamine.net
**SERHII O. KINAKH**	Enamine Ltd	Medicinal Chemistry	78 Chervonotkatska Street, 02094 Kyiv, Ukraine	s.kinakh@mail.enamine.net
**IVAN G. LOGVINENKO**	Enamine Ltd	Medicinal Chemistry	78 Chervonotkatska Street, 02094 Kyiv, Ukraine	i.logvinenko@mail.enamine.net
**KOSTIANTYN P. MELNYKOV**	Enamine Ltd	Medicinal Chemistry	78 Chervonotkatska Street, 02094 Kyiv, Ukraine	k.melnykov@mail.enamine.net
**VICTOR D. HULIAK**	Enamine Ltd	Medicinal Chemistry	78 Chervonotkatska Street, 02094 Kyiv, Ukraine	v.huliak@mail.enamine.net
**IGOR S. TSURUPA**	Enamine Ltd	Medicinal Chemistry	78 Chervonotkatska Street, 02094 Kyiv, Ukraine	i.tsurupa@mail.enamine.net
**KADI SAAR**	University of Cambridge	Cavandish Laboratory	JJ Thomson Ave, Cambridge CB3 0HE	kls78@cam.ac.uk
**BENJAMIN PERRY**	DNDi	Research and Development	15 Chemin Camille Vidart, 1202 Geneva, Switzerland	bperry@dndi.org
**LAURENT FRAISSE**	DNDi	Research and Development	15 Chemin Camille Vidart, 1202 Geneva, Switzerland	lfraisse@dndi.org
**PETER SJÖ**	DNDi	Research and Development	15 Chemin Camille Vidart, 1202 Geneva, Switzerland	psjo@dndi.org
**PASCALE BOULET**	DNDi	Operations	15 Chemin Camille Vidart, 1202 Geneva, Switzerland	pboulet@dndi.org
**SOPHIE HAHN**	DNDi	External Affairs	15 Chemin Camille Vidart, 1202 Geneva, Switzerland	shahn@dndi.org
**CHARLES MOWBRAY**	DNDi	Research and Development	15 Chemin Camille Vidart, 1202 Geneva, Switzerland	cmowbray@dndi.org
**LAUREN REID**	MedChemica Ltd	Senior Computational Chemist	Alderley Park, Macclesfield, Cheshire. SK10 4TG UK.	

**PAUL REES**	Compass Bussiness Partners Ltd			paulhrees@gmail.com
**QIU YU JUDY HUANG**	University of Massachusetts Chan Medical School	Biochemistry and Molecular Biotechnology	55 N Lake Ave, Worcester MA 01655	qiuyu.huang@umassmed.edu
**SARAH N ZVORNICANIN**	University of Massachusetts Chan Medical School	Biochemistry and Molecular Biotechnology	55 N Lake Ave, Worcester MA 01655	sarah.duggan@umassmed.edu
**ALA M. SHAQRA**	University of Massachusetts Chan Medical School	Biochemistry and Molecular Biotechnology	55 N Lake Ave, Worcester MA 01655	Ala.Shaqra@umassmed.edu
**NESE KURT YILMAZ**	University of Massachusetts Chan Medical School	Biochemistry and Molecular Biotechnology	55 N Lake Ave, Worcester MA 01655	Nese.KurtYilmaz@umassmed.edu
**CELIA A. SCHIFFER**	University of Massachusetts Chan Medical School	Biochemistry and Molecular Biotechnology	55 N Lake Ave, Worcester MA 01655	Celia.Schiffer@umassmed.edu
**IVY ZHANG**	Memorial Sloan Kettering Cancer Center	Memorial Sloan Kettering Cancer Center	1275 York Ave, New York, NY 10065	ivy.zhang@choderalab.org
**IVÁN PULIDO**	Memorial Sloan Kettering Cancer Center	Memorial Sloan Kettering Cancer Center	1275 York Ave, New York, NY 10065	ivan.pulido@choderalab.org
**CHARLIE TOMLINSON**	Diamond Light Source Ltd; Research Complex at Harwell	Life Sciences	Diamond Light Source, Harwell Science and Innovation Campus, Didcot, OX11 0DE, United Kingdom; Research Complex at Harwell, Harwell Science and Innovation Campus, Didcot OX11 0FA, United Kingdom	charlie.tomlinson@diamond.ac.uk
**JENNY C. TAYLOR**	University of Oxford	Nuffield Department of Medicine	Wellcome Centre for Human Genetics, Roosevelt Drive, Oxford OX3 7BN, UK	jenny@well.ox.ac.uk
**TRISTAN IAN CROLL**	Cambridge Institute for Medical Research, The	Haematology	Keith Peters Building, Hills Rd, Cambridge CB2 0XY, UK	tic20@cam.ac.uk

	University of Cambridge			
**LENNART BRWEWITZ**	University of Oxford	Department of Chemistry	Chemistry Research Laboratory, 12 Mansfield Rd, Oxford OX1 3TA, United Kingdom	lennart.brewitz@chem.ox.ac.uk
