# Supplementary material for: An in-solution snapshot of SARS-COV-2 main protease maturation process and inhibition
Source: Nat Commun. 2023 Mar 20;14:1545. doi: 10.1038/s41467-023-37035-5 (PMC10027274; doi:10.1038/s41467-023-37035-5)
Supplement: Supplementary file 3 — Reporting Summary [file 41467_2023_37035_MOESM3_ESM.pdf]

nature portfolio

Corresponding author(s): Glaucius Oliva, Andre Schutzer Godoy

Last updated by author(s): Jan 25, 2023

Reporting Summary

Nature Portfolio wishes to improve the reproducibility of the work that we publish. This form provides structure for consistency and transparency in reporting. For further information on Nature Portfolio policies, see our Editorial Policies and the Editorial Policy Checklist.

Statistics

For all statistical analyses, confirm that the following items are present in the figure legend, table legend, main text, or Methods section.

n/a	Confirmed	
☐	☒	The exact sample size (*n*) for each experimental group/condition, given as a discrete number and unit of measurement
☐	☒	A statement on whether measurements were taken from distinct samples or whether the same sample was measured repeatedly
☒	☐	The statistical test(s) used AND whether they are one- or two-sided<br>*Only common tests should be described solely by name; describe more complex techniques in the Methods section.*
☐	☒	A description of all covariates tested
☐	☒	A description of any assumptions or corrections, such as tests of normality and adjustment for multiple comparisons
☐	☒	A full description of the statistical parameters including central tendency (e.g. means) or other basic estimates (e.g. regression coefficient) AND variation (e.g. standard deviation) or associated estimates of uncertainty (e.g. confidence intervals)
☒	☐	For null hypothesis testing, the test statistic (e.g. *F*, *t*, *r*) with confidence intervals, effect sizes, degrees of freedom and *P* value noted<br>*Give P values as exact values whenever suitable.*
☒	☐	For Bayesian analysis, information on the choice of priors and Markov chain Monte Carlo settings
☒	☐	For hierarchical and complex designs, identification of the appropriate level for tests and full reporting of outcomes
☒	☐	Estimates of effect sizes (e.g. Cohen's *d*, Pearson's *r*), indicating how they were calculated

Our web collection on statistics for biologists contains articles on many of the points above.

Software and code

Policy information about availability of computer code

Data collection	ThermoFisher Scientific EPU 2.12,
Data analysis	cryoSPARC v3.2.0, Relion v3.1, CTFFIND-v4.1, SPHIRE-crYOLO v1.6, MotionCor2 v1.4, ChimeraX v1.2.5, Agilent 6530 QTOF, Masshunter B.07.00 (Agilent); ESIprot, ASTRA 7, PyMol v2, Phenix 1.19.2, CCP4 v7.1.017, Molprobity v4.4, 3DFSC Processing Server, Coot 0.9.6, XDS 20220110, Autoproc version 1.0.5, BioRender.com, OriginPro v.9, QualBrowser, OriginPro 2021, Refmac v5.8.0267 , Phaser v2.8.3, Acedrg v222

For manuscripts utilizing custom algorithms or software that are central to the research but not yet described in published literature, software must be made available to editors and reviewers. We strongly encourage code deposition in a community repository (e.g. GitHub). See the Nature Portfolio guidelines for submitting code & software for further information.

Data

Policy information about availability of data

All manuscripts must include a data availability statement. This statement should provide the following information, where applicable:

- Accession codes, unique identifiers, or web links for publicly available datasets
- A description of any restrictions on data availability
- For clinical datasets or third party data, please ensure that the statement adheres to our policy

All data associated with this study are publicly available. All collected movies and polished particles used for final structure determination are available at Electron

Microscopy Public Image Archive (EMPIAR) under the code EMPIAR-10810 [10.6019/EMPIAR-10810]. Cryo-EM maps and structural models are available at Protein Data Bank (PDB) under code 8EY2 [10.2210/pdb8EY2/pdb] and Electron Microscopy Data Bank (EMDB) under the code EMD-28666 [https://www.ebi.ac.uk/emdb/EMD-28666], and X-ray model is available under PDB code 8EYJ [10.2210/pdb8EYJ/pdb]. The structures used in this study are available in PDB database under the codes 7N5Z [10.2210/pdb7N5Z/pdb], 7KPH [10.2210/pdb7KPH/pdb], 7N6N [10.2210/pdb7N6N/pdb], 7K3T [10.2210/pdb7K3T/pdb] and 7DVP [10.2210/pdb7DVP/pdb]. Other data are however available from the authors upon reasonable request. Source data file are provided with this paper for all SEC-MALS and Native mass spectroscopy.

Human research participants

Policy information about studies involving human research participants and Sex and Gender in Research.

Reporting on sex and gender	n/a
Population characteristics	n/a
Recruitment	n/a
Ethics oversight	n/a

Note that full information on the approval of the study protocol must also be provided in the manuscript.

Field-specific reporting

Please select the one below that is the best fit for your research. If you are not sure, read the appropriate sections before making your selection.

☒ Life sciences ☐ Behavioural & social sciences ☐ Ecological, evolutionary & environmental sciences

For a reference copy of the document with all sections, see nature.com/documents/nr-reporting-summary-flat.pdf

Life sciences study design

All studies must disclose on these points even when the disclosure is negative.

Sample size	For structural determination (cryo-EM and x-ray), one sample was used, which is standard for the techniques. We provide a qualitative description of our sample, therefore no quantitative comparisson was performed and no replication was required, so one sample was enough. For native mass spec, two distinct purifications were used, both corroborating the results. For SEC-MALS no sample size calculation was performed, and only one sample for each specimen was used based on availability, and three distinct times were monitored (0, 24h and 48h), all corroborating the conclusions. One sample was enough to reach our conclusions.
Data exclusions	No data was excluded from the analyses. For SEC-MALS experiments, trimers and tetramers peaks were treated as tetramers due to the limitations of resolution.
Replication	For native mass spec, two distinct purifications were used, both corroborating the results. For structural studies, no replication was conducted. For SEC-MALS, only one sample for each specimen was used, and three distinct times were monitored and two concentrations of ligands were used. We provide a qualitative description of our sample, therefore no quantitative comparisson was performed and no replication was required.
Randomization	No randomization was used in this study, as none of techniques required so.
Blinding	Blinding was not relevant to this study, therefore was not used. We provide a qualitative description of our sample, therefore no quantitative comparisson was performed and no blinding was required

Reporting for specific materials, systems and methods

We require information from authors about some types of materials, experimental systems and methods used in many studies. Here, indicate whether each material, system or method listed is relevant to your study. If you are not sure if a list item applies to your research, read the appropriate section before selecting a response.

Materials & experimental systems

n/a	Involved in the study
☒	☐ Antibodies
☒	☐ Eukaryotic cell lines
☒	☐ Palaeontology and archaeology
☒	☐ Animals and other organisms
☒	☐ Clinical data
☒	☐ Dual use research of concern

Methods

n/a	Involved in the study
☒	☐ ChIP-seq
☒	☐ Flow cytometry
☒	☐ MRI-based neuroimaging
